# Using Natural Language Processing and Artificial Intelligence to Explore the Nutrition and Sustainability of Recipes and Food

**DOI:** 10.3389/frai.2020.621577

**Published:** 2021-02-23

**Authors:** Marieke van Erp, Christian Reynolds, Diana Maynard, Alain Starke, Rebeca Ibáñez Martín, Frederic Andres, Maria C. A. Leite, Damien Alvarez de Toledo, Ximena Schmidt Rivera, Christoph Trattner, Steven Brewer, Carla Adriano Martins, Alana Kluczkovski, Angelina Frankowska, Sarah Bridle, Renata Bertazzi Levy, Fernanda Rauber, Jacqueline Tereza da Silva, Ulbe Bosma

**Affiliations:** ^1^ KNAW Humanities Cluster, Amsterdam, Netherlands; ^2^ Centre for Food Policy, City, University of London, London, United Kingdom; ^3^ Natural Language Processing Group, Department of Computer Science, The University of Sheffield, Sheffield, United Kingdom; ^4^ Department of Information Science and Media Studies, University of Bergen, Bergen, Norway; ^5^ Meertens Institute (KNAW), Amsterdam, Netherlands; ^6^ National Institute of Informatics, Chiyoda-ku, Japan; ^7^ Department of Mathematics and Statistics, College of Arts and Sciences, University of South Florida, St. Petersburg, FL, United States; ^8^ Equitable Development and Resilience Research Group, Institute of Energy Futures, College of Engineering, Design and Physical Science, Brunel University London, Uxbridge, United Kingdom; ^9^ Text Mining Solutions Ltd., York, United Kingdom; ^10^ Department of Physics & Astronomy, Faculty of Science and Engineering, The University of Manchester, Manchester, United Kingdom; ^11^ University of São Paulo, São Paulo, Brazil; ^12^ International Institute of Social History (KNAW), Amsterdam, Netherlands

**Keywords:** natural language processing, semantic web, computational recipe analysis, food history, interdisciplinary, recommender systems, food science, food computing

## Abstract

In this paper, we discuss the use of natural language processing and artificial intelligence to analyze nutritional and sustainability aspects of recipes and food. We present the state-of-the-art and some use cases, followed by a discussion of challenges. Our perspective on addressing these is that while they typically have a technical nature, they nevertheless require an interdisciplinary approach combining natural language processing and artificial intelligence with expert domain knowledge to create practical tools and comprehensive analysis for the food domain.

## Introduction

Today’s big societal challenges are increasingly analyzed from a data-driven perspective (van Veenstra and Kotterink, 2017), while the universal pervasiveness of food and its inherent multidisciplinary nature (Deutsch and Miller, 2007) enable it as an accessible window into every culture and time period. Many global challenges are directly related to food, nutrition, and sustainability.^1^ At least 6 of the UN’s Sustainable Development Goals involve food (UN, 2015). The food system is linked to 30% of total greenhouse gas emissions (Mbow et al., 2019), and healthcare costs are increasing due to diet-related issues (Schulze et al., 2018; Branca et al., 2019); 60%+ of adults in the United Kingdom and United States are now obese or overweight (WHO, 2020). Food is also central to many countries’ economies (11% of total employment in the US and the Netherlands (Hausmann et al., 2014; FAO, 2019)) and cultural heritage (Richard and Coste, 2020). The ability to research food and recipes can help us address the challenges of sustainable and healthy eating in diverse cultural contexts, particularly given the current need to move to a more plant-based diet (Willett et al., 2019).

However, making the information on food accessible is far from trivial. Analysis of digitized or digital recipes is a new and upcoming field of research, with publications linked to nutritional and health studies (Reinivuo et al., 2009; Trattner et al., 2017), computational linguistics (Jurafsky, 2015), computational gastronomy (Jain et al., 2015), shopping (Aiello et al., 2019), allergen detection (Alemany-Bordera et al. 2016; Amato and Cozzalino, 2020), and the Semantic Web (Haussmann et al., 2019). Several research challenges are at the crossroads of data engineering, intelligent food, and cooking recipes, as discussed at the recent IEEE DECOR@ICDE workshop series (Andres et al., 2020). Furthermore, contemporary recipe analysis is underdeveloped in terms of links to sustainability—beyond publications by Reynolds and collaborators (Reynolds, 2017, Reynolds, 2019; Quadros et al., 2019; Reynolds et al., 2019), Andres and collaborators (Andres et al., 2018; Fritzen et al., 2018; Andres et al., 2019; de Toledo et al., 2020; Toledo et al., 2020), Asano and Biermann (2019), and Herrera (2020).

We suggest that the reason this problem has not been addressed in an integrated manner is partly due to the complexity of linking environmental impact databases to food terminology, which is time-consuming without artificial intelligence (AI) and natural language processing (NLP) tools. It is only in the last few years that these methods have been applied to combining recipes, food texts, and other environmental, nutritional, and economic databases, but this work is still incipient.

In this article, we encourage an interdisciplinary approach to the exploration of nutrition and sustainability. We highlight challenges and opportunities of using AI to analyze the food domain through recipes and present use cases that form the basis of a collaborative movement to provide a multifaceted and data-driven analysis of nutrition and sustainability. First, we explore issues around collecting and integrating food, nutrition, and sustainability data. Second, we review the NLP and other AI methods currently employed in linking and analyzing these data sources. We conclude by discussing how such techniques can be used to engage and translate food challenges to stakeholders and forecast possible future applications such as novel kinds of recommender systems that encourage positive behavioral change.

### Challenges With Food, Recipe, Nutrition, and Sustainability Data

A major challenge with recipe datasets is that no standard online collections exist yet due to restrictions in terms of use, language, etc. Previous research often uses proprietary materials through APIs including crawls from online recipe collections and databases such as epicurious.com, allrecipes.com, RecipeDB, CulinaryDB, Hawa World, TarlaDalal.com, and chefkoch.de (Shidochi et al., 2009; Ahn et al., 2011; Teng et al., 2012; Ahnert, 2013; Jain et al., 2015; Kusmierczyk et al., 2015; Bogojeska et al., 2016; Tallab and Alrazgan, 2016; Kikuchi et al., 2017; Sajadmanesh et al., 2017; Bagler and Singh, 2018; Chang et al., 2018; Min et al., 2018; Asano and Biermann, 2019; Batra et al., 2019; Trattner and Elsweiler, 2019; Herrera, 2020; Sharma et al., 2020). To be useful in practical applications, these untapped sources require structuring, linking, and analysis via NLP techniques.

High-quality nutrition databases are compiled by multiple global organizations (e.g., Louie et al., 2016; FAO/WHO, 2020; UK, 2020; USDA, 2020; EFSA, 2020; and RIVM, 2020) for their respective geographies. However, each has its own coding standards and hierarchy, making them inflexible, and thus time-consuming and difficult to combine, compare, or integrate. For example, the USDA has a large archive of its national nutritional recommendations organized chronologically, allowing researchers to investigate changes in nutritional recommendations across time. The FAO, on the other hand, organizes its data around global food systems with a strong mission to fight malnutrition and hunger and incorporate global UN programs. AI tools are already being used to link and reconcile databases, fill data gaps, and establish common ontological frameworks (Eftimov et al., 2016; Eftimov et al., 2017; Ispirova et al., 2017; Dooley et al., 2018; Ispirova et al., 2019; Ispirova et al., 2019; Popovski et al., 2019), but the problem is not trivial.

Sustainability data are less coherent and not consistently available. Over the last twenty years, databases of aggregated meta-analysis of life cycle analysis (LCA) studies have emerged, providing sustainability information linked to specific food products (e.g., climate change, water, land use, or biodiversity impacts). Additionally, there are paywalled or consultancy LCA databases. Although most of these follow standards (e.g., ISO14040/44 or BSI-PAS 2050), key aspects that influence the results, such as the scope of the study (e.g., cradle-to-gate and cradle-to-grave), functional units used (e.g. mass, volume, and calories), and assumptions made are not always clear or well-documented, make it difficult for nonexperts to interpret, use, and apply this content to more comprehensive studies like those about healthy and sustainable food. Recently, Ghose et al., (2019) and Ghose (2020) proposed NLP methods for semantic investigation of LCA databases. However, while the sustainability data might be available for individual ingredients, it is still rare for entire recipes. The computation of a recipe’s sustainability data, such as its carbon footprint, includes taking into account the combination and volumes of different ingredients.

It is clear that while a number of knowledge sources are available, a major challenge is that there are no (or limited) direct links between and among nutrition, sustainability, and recipe databases, with differing levels of data granularity even in databases of the same type. Furthermore, there are often linguistic, conceptual, and terminological gaps between the different kinds of knowledge sources, and while in principle, the immense amount of data allows for very detailed views of specific knowledge domains; the lack of any interconnecting framework makes this information largely incommensurable across different dimensions.

Furthermore, most analysis is limited to small-scale manual efforts that do not have a temporal aspect, with little connection between quantitative and qualitative methods. Linking approaches in the Semantic Web sphere are, in some cases, more well-developed but mostly do not relate to the sustainability aspect, and they are more targeted at shopping and healthy recipe applications. Current applications typically also focus on digital data that are already at least semistructured and do not require complex NLP.

### Challenges for NLP in Computational Recipe Analysis

Contemporary recipe analysis is a well-researched field (Reinivuo et al., 2009; Trattner et al., 2017). However, once recipes do not come from the same source document or are not digitally born, automatic recipe analysis becomes a complex problem for language technology tools (van Erp et al., 2018).

When analyzing older data, artifacts from the digitization process may insert errors in the text and units of measurement and language usage may differ according to the source, region, or time period. This needs to be addressed first to enable comparison between recipes over time and space. In this section, we present a use case on automatically analyzing sugar quantities from historical apple pie recipes to illustrate some of the challenges.

We analyzed apple pie recipes for Dutch, American, French, and German and found that differences in coverage of the sources, data access via the different portals,^2^ and classification of recipes (as not all retrieved articles mentioning apple pie are indeed recipes) required tailoring the tools to each resource. Artifacts of the digitization process, such as Optical Character Recognition^3^ errors hamper these processes, as not all characters are recognized correctly, rendering parts of a sentence or even entire documents unreadable (e.g., “% Pfund Zucker” for “¼ Pfund Zucker”). This is as yet an unsolved problem (van Strien et al., 2020).

Quantities can be expressed by numerals and fractions or spelled out, and units are expressed as metric, imperial, or other measurements such as teacups. Here, not just conversion tables but contextual knowledge are needed as, for example, teacups vary in size between North American and Europe. Quantities are also not always specified (e.g., “Honig oder Zucker nach Süße der Äpfel und Gusto” or “2 sucre”) or are difficult to assess when recipes use preprocessed ingredients such as compote and/or ready-made pastry, with unknown sugar content. These problems often also exist with modern recipes.

Additionally, often recipes do not mention the number of portions produced, and it is unknown how often people eat pie and how big a portion they typically eat. Therefore, we could not automatically normalize these to a “per person” or “portion” quantity. One could use a typical portion size, calculated from similar recipes, or have a portion size based on calories, but this requires further analysis and transformation.

Even for contemporary, digital-born recipes enriched with structured data, quantity extraction is not trivial. In analyzing recipes from the American site Allrecipes.com and its British site Allrecipes.co.uk, we found that while both were ostensibly from the same organization, the webpage structure of the two domains was quite different; thus, different analysis scripts had to be created for each. It is not always easy to retrieve the publication dates of these recipes, making it difficult to correctly assess the recipe’s publication date and to use it in recipe trend analysis.

### Challenges of Analyzing Contemporary Recipes for Nutrition

In the field of nutrition, the presentation and analysis of recipes is usually done through “technical preparation sheets.” Traditionally, these sheets contain a list of ingredients, culinary techniques, preparation times of the dishes, necessary equipment, and portion sizes. They also quantify the calorific value and macro (and micro) nutrients of each recipe. This latter quantification is carried out by manually linking to food composition tables or automatically with specific nutrition software (Tufts University, 2020). This not only enables the standardization of recipe preparation but also acts as a support tool for the composition of nutritionally balanced menus (see Akutsu et al., 2005). Another way of presenting and analyzing recipes in terms of nutrition is through the Nutriscore scale (Chantal et al., 2017), a European index for foods that was developed as part of the French Health Law in 2016. Its goal is to improve the nutritional information shown on food packages to help consumers make healthier purchases. NLP techniques are currently scarcely adopted by the professional nutritionist community, who rely largely on manual techniques. There are also commercial APIs (e.g., provided by Edamam.com or Spoonacular.com) that offer nutrition integration into recipes using NLP. These have found wide customer bases but are not widely used in the nutrition-practitioner community.

Similar critical NLP issues exist when linking recipes to nutritional data. First, many recipes are still only found in printed and handwritten books, and modern recipe books have much irregular formatting. Even modern recipes contain vernacular ingredients, cooking methods and cooking temperatures, and units (e.g. 25 g sprigs of mint, 1 slice of large kohlrabi, a bunch of coriander, 3 baby Brussels sprouts, and 1 pinch salt), and developing ways to normalize and interpret these is time-consuming. Likewise, geographic differences may cause ambiguity (e.g., United States and United Kingdom tablespoon size). Methods to handle noisy recipe data and process it efficiently have been discussed (Trattner et al., 2019), with pipelines used to predict nutrient values of recipes (Trattner and Elsweiler, 2017; Rokicki et al., 2018).

### Challenges of Analyzing Recipes for Sustainability

Contemporary recipe analysis is underdeveloped in terms of links to sustainability. One method of assessing sustainability of a recipe is to link it to an existing quantified environmental measure. Multiple groups have now used some NLP to map specific measures such as greenhouse gas emission (GHGE) and nutrient variables to standard food classifications such as FoodEx2 (Eftimov et al., 2017; Mertens et al., 2019; Quadros et al., 2019; Reynolds et al., 2019). Additional web visualization tools examining the GHGE of foods have been developed for manual recipe analysis (US EPA, 2020; The Vegan Society, 2020).^4^ ^,^ ^5^ One of the most advanced web tools is the NAHGAST Online Tool, which can provide economic, health, social, and environmental (material footprint, carbon footprint, water use, and land use) footprints of user submitted recipes (Speck et al., 2020). This has had 1,509 user-submitted recipes in the first research phase, with focus on empowering Out-of-Home Catering Sector users to reduce their impacts. Despite the proliferation of these web-based tools, for recipe exploration, little advantage has been taken of the full range of NLP’s capabilities.

**FIGURE 1:**
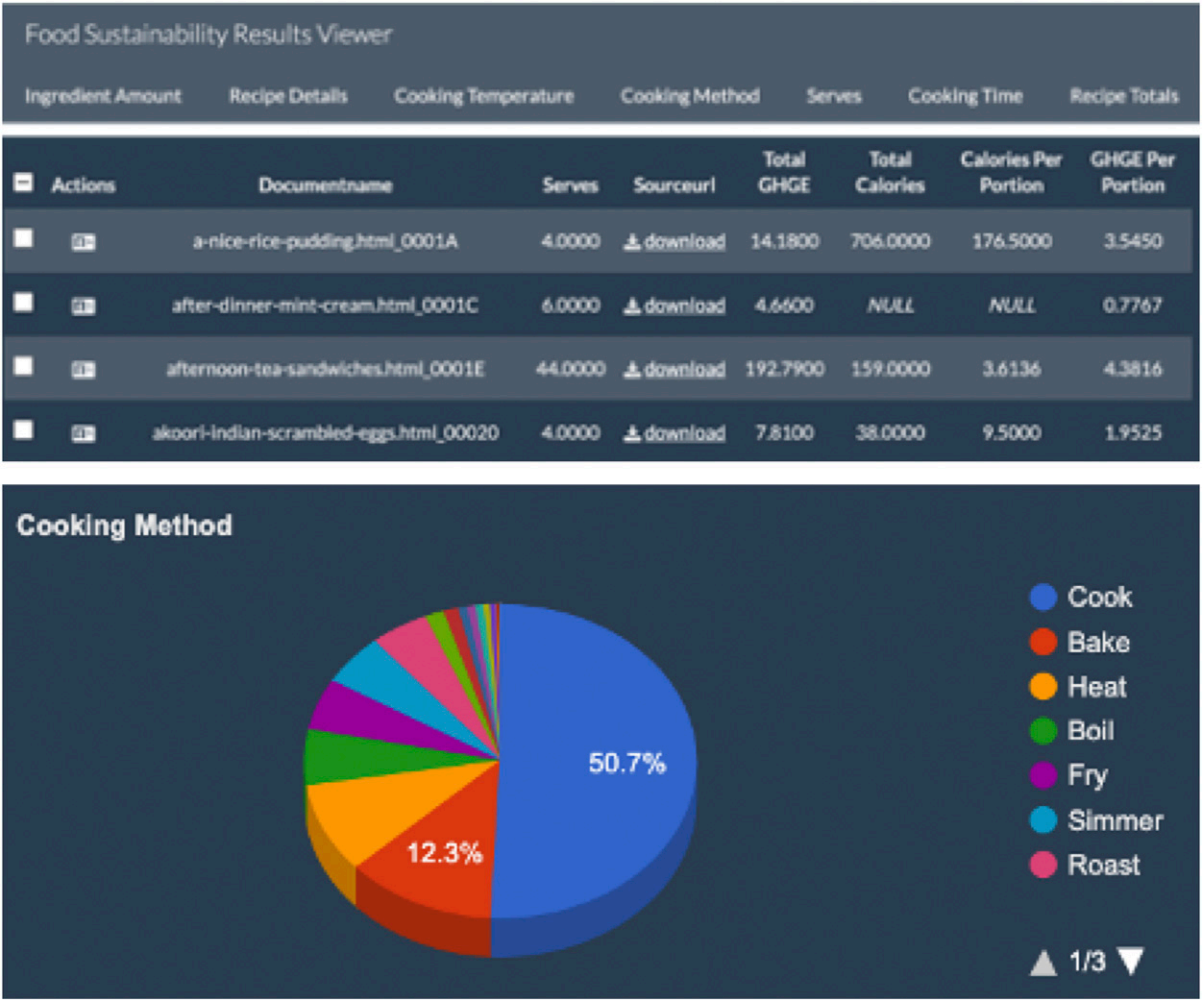
Screenshots from the pilot food sustainability results viewer produced by Text Mining Solutions Ltd.

Reynolds and collaborators (in unpublished pilot work) collaborated with Text Mining Solutions Ltd. to map sustainability information to recipes. This pilot project used the GATE NLP toolkit (Cunningham et al., 2002), as the framework for extracting information extraction. They then applied environmental impact and calorie data to each ingredient per portion and calculated an overall figure for the recipes’ footprints to understand the environmental impact and trade-offs of the recipes. Text Mining Solutions Ltd. also created a web visualization tool (see Figure 1) to enable citizen engagement and interactive exploration of trade-offs between recipes, sustainability, and nutrition.

Concurrently, Andres and collaborators (Andres, 2018; Andres et al., 2018; Andres et al., 2019; de Toledo et al., 2020; Toledo et al., 2020) created the CROPPER service (CaRbon fOotprint reciPe oPtimizER). CROPPER improves an input recipe by updating its ingredients and cooking procedures to reduce its carbon footprint while keeping it savoury. CROPPER is part of the CRWB group research^6^ created in memory of the cook Nicole Andres (1942–2016) to manage her 60-year cooking recipe collection legacy and enhance it for data science research.

Asano and Biermann (2019) used NLP-driven recipe analysis to examine dietary transitions toward sustainable diets but did not link this to environmental impacts, examining instead the number and composition of vegetarian and vegan recipes submitted. Finally, Herrera (2020) used a recommender system to minimize food waste and recommend recipes using (organic) locally grown food. Interestingly, this provides a link between recipes, supply chain, and modes of production.

When discussing recipe analysis, the environmental impacts of cooking cannot be ignored, accounting for as much as 61% of total emissions associated with specific foods (Frankowska et al., 2019). However, only de Toledo et al. (2020) provided a published framework for the combination and extraction of cooking data from recipes. This calculation needs additional data including cooking time, the energy consumption of home appliances (per kW h), and the carbon emissions (per kW h) related to the specific energy grid. To date, there has been no real-world application that calculates the environmental impacts of recipes, though various strands of research are underway from Reynolds, Andres, and Trattner, along with collaborators (see the “Introduction” section).

### Future Directions

Our research opens up an avenue of new possibilities for food personalization and engagement in shifts toward healthy sustainable diets and cooking.

In particular, recommender technology can be integrated into current recipe websites and apps to improve support for users who wish to adopt healthier and/or more sustainable eating habits. A disadvantage of such personalized systems is that they typically reinforce existing eating habits (Starke, 2019), encouraging users to buy more of the same products rather than try healthier alternatives, and even so-called “persuasive” agent-based recommenders may still be based on existing lifestyle choices and social network activity (Palanca et al., 2014). NLP-based methods not only make it easier to compute the healthiness or sustainability of recipes but could also allow the design of personalized interventions that are rapidly explainable, updatable, and deployable, highlighting different categories that cater to different eating goals, such as health or sustainability. Additionally, Fritzen et al. (2018) have begun to focus on the integration of collective intelligence and AI via social networks to evaluate the gap between citizens’ dish expectations and tasting experiences.^7^

A further limitation of current NLP recipe analysis is related to geographically contextualizing diets, nutrients, and food footprints, which is critical for global relevance. Current nutrient and environmental impact databases are not detailed enough to provide analysis and recommendations at different geographic levels (e.g. Western Europe and East Asia have very different requirements).

If adopted and implemented correctly, recipes analyzed and contextualized with NLP and linked to recommender systems will be useful to the general public as well as providing an analytical tool for specialists (including nutritionists, historians, chefs, educators, and policymakers). Enhancing recommender systems with multimedia capabilities (taste, texture, and smell) (Ghinea et al., 2011) could enable a better comprehension of recipes and target dishes. The food industry and supermarkets are obvious adopters of this technology through the Internet of Food Things;^8^ while archives and libraries can use this technology to engage citizens with their collections. Government and nongovernment organizations can use this technology to monitor gastronomy, food culture, and dietary patterns and form comprehensive and adaptive policies.

## Conclusion

Our perspective is that food and recipe research to help solve health and sustainability issues needs to be addressed in an interdisciplinary fashion, integrating NLP and other AI techniques with historical food research, food science, nutrition, and sustainability expertize. As outlined in this paper, multiple technical challenges still need to be solved. However, a purely technical approach is not sufficient: despite numerous advances in NLP, technology needs to be tightly interwoven with expert knowledge, highlighting the need for engagement with a wider interdisciplinary community. The collaborative work demonstrated in this paper shows that the combined viewpoints and expertize can make extremely encouraging steps toward addressing and resolving these critical issues.

## Data Availability

The original contributions presented in the study are included in the article, and further inquiries can be directed to the corresponding author.

## Footnotes

1 Food analysis was an essential entry point to explore cultures in early anthropological works, for example, as a proxy to understand material cultures, material practices, taboos, and social relations and rituals. Later, food has become an important focus of analysis to theorize colonialism and the machinery of empire, globalization, and more recently, urbanization and political ecology.

2 For Dutch recipes, we used the Delpher.nl portal maintained by the Dutch National Library, for American, the Chronicling America portal maintained by the US Library of Congress, for French, Gallica maintained by the Bibliothèque Nationale de France, and for German, the Europeana Newspapers portal.

3 Optical Character Recognition is the process of automatically converting text from an image (scanned) to machine readable format.

4 Take a Bite out of Climate Change (2020). Available at: https://www.takeabitecc.org/calculator.html (accessed October 23, 2020).

5 Greenhouse Gas Footprint Calculator. Available at: https://www.tuco.ac.uk/ghgcalculator/index.html (accessed October 23, 2020).

6
https://www.researchgate.net/project/Cooking-Recipes-Without-Border-Research-group.

7
https://www.researchgate.net/project/Big-Data-LOD-benchmark-meets-Intelligent-Food-and-Cooking-Recipe.

8
https://www.foodchain.ac.uk/
